# “Hey Amir, How Are You REALLY Doing?”: Participant Perspectives of a Peer-Based Suicide Prevention Campaign for Men

**DOI:** 10.1177/15579883231209189

**Published:** 2023-10-30

**Authors:** Paul Sharp, Patricia Zhu, John S. Ogrodniczuk, Zac E. Seidler, Michael J. Wilson, Krista Fisher, John L. Oliffe

**Affiliations:** 1 School of Nursing, The University of British Columbia, Vancouver, BC, Canada; 2 Department of Psychiatry, The University of British Columbia, Vancouver, BC, Canada; 3 Orygen, Parkville, VIC, Australia; 4 Centre for Youth Mental Health, The University of Melbourne, Melbourne, VIC, Australia; 5 Movember, Melbourne, VIC, Australia; 6 Department of Nursing, The University of Melbourne, Melbourne, VIC, Australia

**Keywords:** men’s mental health, suicide prevention, peer support, masculinity

## Abstract

Suicide is a major public health concern and leading cause of death among men in Canada. This study reports the feasibility and acceptability of Buddy Up, a peer-based suicide prevention campaign for men. A mixed-methods approach was used to analyze respondent survey questionnaires (*n* = 48) and individual participant interviews (*n* = 19) collected from campaign users. Survey respondents reported that they enjoyed their involvement in the campaign (92%), were more confident to talk with men about mental health and suicide (95%), and would recommend Buddy Up to others (95%). Qualitative interviews were thematically analyzed to develop three inductively derived themes: (a) Engaging men with relatable masculine content and design: “Buddy Up really spoke to them in their language,” highlighting the importance of understanding and working with gendered practices and motivations to legitimize and motivate involvement in suicide prevention; (b) Leveraging campaign participation to initiate conversations and promote mental health: “It gives men language and license to start asking questions,” revealing ways in which participants utilized Buddy Up to negotiate and norm checking-in to promote men’s mental health; and (c) Driving new masculine cultures: “We start every meeting with a mental health moment,” identifying how participants fostered healthy milieus for disclosing mental health challenges with teamwork and preventive action under the banner of Buddy Up. The study findings support the feasibility of Buddy Up and highlight the acceptability of peer-based approaches to mental health promotion. The findings can also empirically guide future efforts for systematically building men’s peer-based suicide prevention programs.

## Introduction

Suicide persists as a leading cause of death worldwide (World Health Organization, 2021), with men accounting for more than 75% of all suicide deaths in Canada (Statistics Canada, 2022). Suicide risk is complex and multifaceted with factors known to elevate risk including depression, grief, substance use, social isolation, and illness (Centres for Disease Control and Prevention, 2022). Men’s disproportionally high suicide rates have been characterized as a “silent crisis,” and gender-related factors are often implicated in men’s reticence for help-seeking and low uptake of professional services (Ogrodniczuk et al., 2023). Traditional masculine norms, including stoicism, self-reliance, and restrictive emotionality, can contribute to a reluctance to seek professional help for mental health challenges (Seidler et al., 2016). Men’s mental health challenges can manifest diversely, including a range of externalized symptoms (e.g., anger, substance misuse, excess sexual activity; Oliffe et al., 2019), which diverge from commonly used diagnostic criteria for depression (Rice et al., 2013) and anxiety disorders (Fisher et al., 2021). As a result, men’s mental health challenges may go undiagnosed, misrepresented, and overlooked (Rice et al., 2022). Among men who have sought professional help, dropout rates are high (45%), as services may not be designed or delivered to align with men’s values and preferences for support (Seidler et al., 2021). As an alternative, many men prefer informal sources of help-seeking, such as via friends, family members, or colleagues (Ramirez & Badger, 2014). To combat high rates of male suicide and barriers to men’s help-seeking, public health and peer-based efforts are needed that engage and retain men with tailored mental health promotion and suicide prevention programs.

Suicide prevention campaigns have demonstrated promising results in a variety of contexts (e.g., increased awareness and reduction in suicide attempts) and as such are considered a cost-effective and effective approach for reducing the incidence of suicide and disability due to depression (Lebenbaum et al., 2020; Vasiliadis et al., 2015). Suicide prevention campaigns for men are emergent within this context, with multifaceted campaigns targeted at awareness, providing education, and/or training communities in early detection and intervention (Struszczyk et al., 2019). In doing so, there has been an interest in peer- and community-based efforts to involve and engage men (Oliffe et al., 2019). Peer-based interventions offer a promising avenue and adjunct to professional treatment with much potential for reaching and engaging men in mental health promotion and suicide prevention (Gillard, 2019). Peer support can offer a unique sense of camaraderie, understanding, and empathy that traditional mental health services may not provide (Lefkowich & Richardson, 2018; Robinson et al., 2015). Peer support can also help to transform traditional masculine norms in destigmatizing mental illness to norm reciprocity and mutual help (Vickery, 2022). By affirming men’s experiences, peers can also enhance social connectedness and belonging to reduce suicidality risk (McKenzie et al., 2018).

**Figure 1.**
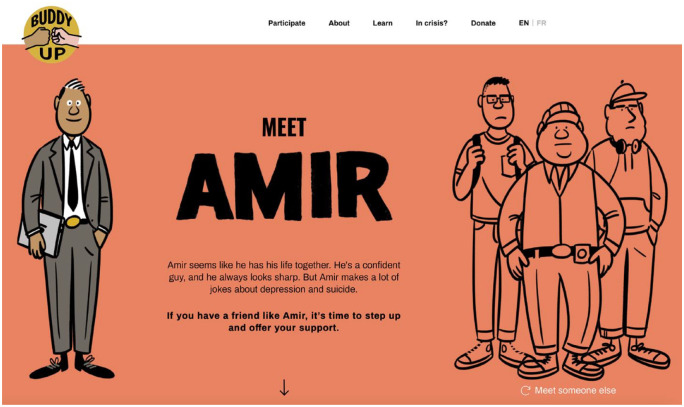
Characters Featured on the BuddyUp.ca Homepage

The current study explores participants’ experiences and perspectives of Buddy Up, a men’s suicide prevention campaign developed by the Center for Suicide Prevention in Calgary, Alberta, Canada. Buddy Up was developed through a co-production process involving a community advisory committee and focus groups with 60 men to design and disseminate targeted advertising, promotional materials, and educational resources. The campaign features nine characters (e.g., businessman, student athlete, and farmer) that depict men experiencing various mental illness symptoms (Figure 1 Characters featured on the BuddyUp.ca homepage). The campaign encourages men to “Buddy Up” by looking for signs of distress in their peers and offering support (“Hey Amir, how are you REALLY doing?”). Individuals (i.e., any gender) register for free to be a Buddy Up Champion and promote men’s mental health and encourage others to get involved. Champions can order promotional materials (e.g., stickers, posters, etc.), intended to be placed around the home, workplace, or community, that raise awareness and promote conversations about men’s suicide prevention. The campaign website includes educational information (e.g., warning signs and gendered risk factors) and resources (e.g., infographic with steps for providing peer support). During the month of June (Buddy Up Month), individuals and groups are invited to complete challenges (e.g., exercise with a buddy) for the chance to win prizes. The current study examined the feasibility and acceptability of Buddy Up to describe key factors that contribute to men’s engagement with suicide prevention campaigns more broadly.

## Method

Mixed methods including a survey questionnaire and individual interviews were used to examine the feasibility and acceptability of Buddy Up. A convergent design comprising qualitative and quantitative data was collected post-campaign and jointly reported to describe participant perspectives and experiences of the campaign. This approach was chosen to garner rich insights into the feasibility regarding individual participation, campaign utility and utilization, and overall satisfaction and acceptability. Ethics approval for this study was provided by the University of British Columbia (ID:H22-02240).

### Data Collection

#### Online Survey

Buddy Up participant survey data were collected online by the Center for Suicide Prevention and the research team conducted a secondary analysis in deciding the qualitative questions to be asked of campaign users. Participants were invited via a campaign mailing list of approximately 650 registered users in June 2022. Specifically, individuals were invited to share their perspectives and experiences of the Buddy Up Campaign in a survey questionnaire requesting respondents’ characteristics, use of campaign features and materials, and overall satisfaction with the campaign. The survey took approximately 15 min to complete and included four-point Likert-type scales (e.g., “Overall, I was satisfied with the Buddy Up campaign”), with 4 indicating the most positive rating on each item, and open-ended response questions (e.g., “What suggestions do you have to improve the campaign?.” Participants who completed the questionnaire were entered into a draw for a $20 e-gift card.

#### Individual Interviews

An email invitation was sent via the campaign mailing list to invite potential participants to complete an individual interview regarding their experiences and perspectives of Buddy Up. Interested individuals contacted the project coordinator via email and were provided a link to complete an e-consent and demographics questionnaire. Once consent was obtained, participants completed a 45- to 60-min Zoom interview at a mutually convenient time. All interviews were conducted by a post-doctoral researcher with expertise in men’s mental health and experience in qualitative research. A semi-structured interview schedule was developed based on feasibility parameters including motivation for participating, acceptability of the campaign and content, and experiences with promoting men’s mental health and suicide prevention (Supplemental Appendix A). Interviews were video and audio recorded, transcribed verbatim, and identifiable information was removed to ensure anonymity and confidentiality prior to analyses. Participants were sent a $20 e-gift card after the interview in recognition of their time and contribution to the study.

### Data Analysis

Quantitative data (online survey responses) were analyzed using IBM SPSS and data are reported as means and frequencies. Qualitative data were thematically analyzed to examine participants’ experiences and perspectives of Buddy Up (Braun & Clarke, 2006). Transcripts were read and re-read (**PS, PZ**) by coauthors to allow for detailed discussion of the data, coding, and interpretation. Codes were inductively generated by identifying key features of participants’ experiences relevant to the study objectives (Braun & Clarke, 2019). For example, data pertaining to campaign utilization, participant motivations, and/or the gendered dimensions of peer support were data-driven codes used to fracture and analyze the data. Coded data were then examined for similarities, grouped, and refined to derive and differentiate themes that reflected participants’ experiences and perspectives of Buddy Up. Descriptive summaries of the narrative data, along with representative quotes and researcher-assigned participant pseudonyms, are included for each theme.

## Results

### Participant Characteristics

A total of 48 respondents completed the survey questionnaire with a mean age of 42.29 (*SD* = 12.71, range 21–69) years. Most respondents had previous experience with suicide prevention (*n*=37, 77%) and were women (*n* = 29, 60%). Interview participants’ (*n* = 19) mean age was 47.89 (*SD* = 14.7, range 24–70) years, and they were predominantly men (*n* = 15, 79%), lived in Alberta, Canada (*n* = 14, 74%), and were employed for wages (*n* = 13, 68%). Approximately half of interview participants (*n* = 9; 47%) were employed or volunteered for mental health related work within health care, government, or non-profit organizations. Most interview participants had experience promoting Buddy Up for at least two annual campaigns (*n* = 14, 74%). Participant characteristics for interview participants are presented in Table 1.

**Table 1.** Interview Participant Characteristics

Characteristics	Interview participants (*N* = 19) *n* (%)
Age	
Mean (*SD*; range: 24–70 years)	47.89 (14.7)
Gender	
Men	15 (78.9)
Women	4 (21.1)
Registration year	
1 Year	5 (26.3)
2 Years	10 (52.6)
3 Years	4 (21.1)
Organization/group size (*n* = 6)	
Small (0–100)	3 (15.8)
Medium (100–500)	1 (5.3)
Large (500+)	2 (10.5)
Location	
Alberta	14 (73.7)
British Columbia	2 (10.5)
Other	3 (15.8)
Education	
Some or all high school	1 (5.3)
Some or all of college	1 (5.3)
Technical diploma or trade	2 (10.5)
Bachelor’s Degree	9 (47.4)
Master’s Degree	6 (31.6)
Cultural Background	
European	14 (73.7)
First nations or Indigenous	2 (10.5)
Other	2 (10.5)
Prefer not to say	1 (5.3)
Income ($CAD)	
<$50,000	5 (26.4)
$50,000–$99,999	4 (21.0)
>$100,000	7 (36.8)
Prefer not to say	3 (15.8)
Employment status	
Employed	13 (68.4)
Student (full-time)	1 (5.3)
Looking for employment	2 (10.5)
Retired	3 (15.8)

### Survey Findings

The majority of survey respondents reported that they were satisfied with the campaign (*n* = 34/37; 92%), enjoyed their involvement (*n* = 35/37; 95%), and would recommend Buddy Up to others (*n* = 35/37; 95%). Open-ended survey responses identified “straightforward messaging,” “easy and accessible language,” and “relatable branding and graphic” as important campaign features. Survey participants reported engaging in an average of 5.8 (*SD* = 3.05; range 0–12) campaign activities including telling others about the campaign via email, text, or word of mouth (*n* = 37/48; 77%); reading resources on the Buddy Up website (*n* = 36/48; 75%); putting up posters (*n* = 34/48; 71%); and using a Buddy Up sticker or air freshener (*n* = 33/48; 69%). Participants reported initiating a range of conversations as a result of Buddy Up including “casual discussions with friends and family,” “one-on-one support with a friend,” and “talking to a colleague about their anxiety following a break-up.” A majority reported that they were more confident to talk with men about mental health (*n* = 35/37; 95%) and suicide (*n* = 35/37; 95%) after participating in the campaign and that the campaign had helped prepare them to talk with a man who was considering suicide (*n* = 33/37; 89%). However, some survey respondents (*n* = 7/44; 16%) indicated that they were not ready to have a conversation with someone about suicide. Recommendations to improve the campaign included “more guidance for how to initiate activities,” “tools on how to start the conversation with someone who is not close to you” and “information on how to respond to statements like ‘I’m not suicidal so this has nothing to do with me.’”

### Qualitative Themes

Three overarching themes were identified that reflected participants’ experiences and perspectives of Buddy Up: (a) Engaging men with relatable masculine content and design: “Buddy Up really spoke to them in their language”; (b) Leveraging campaign participation to initiate conversations and promote mental health: “It gives men language and license to start asking questions”; and (c) Driving new masculine cultures: “We start every meeting with a mental health moment.” While connected, themes are presented separately to provide a detailed analysis of three inductive components underpinning the campaign’s feasibility.

#### Engaging

Men With Relatable Masculine Content and Design: “Buddy Up Really Spoke to Them in Their Language”.

Interview participants highlighted how the design and messaging were appealing to men in a range of settings and contexts. James, a 52-year-old health service provider who promoted Buddy Up to men working in male-dominated, resource-based industries, explained:

I thought that Buddy Up really spoke to them in their language. The characters that they used, the simplicity of the message, and the language they used, it was really attractive. We were out at a drilling rig on a platform, and one of the guys had a Buddy Up sticker on his helmet along with a whole bunch of other inappropriate stickers. But you know, it was kind of the defining moment for me. These guys know what it’s about. They’re not putting it on there because it’s just another sticker. I think it really served the purpose of breaking through some barriers.

James alluded to the challenges of engaging men in settings where traditional masculine values are often staunchly upheld. Highlighted was men’s covert but intentional support of the campaign and resources. That the Buddy Up materials were prominently displayed at the worksite suggested that the imagery and messaging were acceptable and resonated with many men. James also referred to the Buddy Up characters, which feature prominently on the campaign website and print posters, depicting men of diverse ages, backgrounds, and circumstances experiencing common mental health challenges. Herein, men are called to “step up and offer support” to other men in their lives who may be struggling. These relatable characters and calls to action might be understood as increasing awareness about distress in men as well as norming men’s provision and receipt of peer support for mental health challenges. In this way, men’s engagement with the campaign was leveraged according to masculine protector and provider identities. For many, including Rick, a 56-year-old project manager who promoted Buddy Up in his workplace, the desire to help others was key to their campaign involvement:

It started to show up on our internal webpage and I thought, “this is something that I should participate in.” I have a feeling that due to some things I’ve been through, that I have a duty to give back, and a duty to help. That’s easier said than done because I’m not sure what to do but this came up and it was very much mapped out. There’s material, there’s support, and I thought “Well what better thing to jump on than this.”

Rick explained how Buddy Up provided him opportunities to share his vulnerabilities and the knowledge that he had gained from his personal experiences to help others who may be struggling. For many men, providing support and helping others was strength-based and asset-building, and participants found value and purpose in promoting the peer-support messages via the campaign. Several participants noted personal benefits from their involvement in Buddy Up that stemmed from contributing to a cause to help others and provide peer support. Indeed, Mark, a 35-year-old man currently looking for employment, discussed his involvement in Buddy Up:

My mental health is being challenged right now. Given the circumstances with the pandemic, my separation, and not knowing what is going to happen with the kids or our matrimonial home, which is likely getting sold. It was almost like just having it [Buddy Up] as a resource to keep me grounded in something that felt meaningful. Instead of sticking to my own personal challenges, at the time I felt like I wasn’t really getting out of those, something like the Buddy Up campaign could allow me to extend beyond myself and help other people. It felt like a connection to the world outside, the world beyond me and my own space. Getting that sense of greater purpose was a boost which increased my own mental wellness.

For Mark, the mental health challenges stemming from his disrupted relationships and interruption of paid work contributed to a loss of control which drove him to seek purpose through his campaign participation and reciprocal peer relationships. Mark spoke to the benefits of giving at a time when he was losing so much—as a distraction in part, but also as a force for grounding and solace in certainty. In recognizing one’s own challenges and norming those states, men can redirect their distress as energy to authentically prioritize and contribute to helping others who are struggling.

Findings in this theme underscore the importance of understanding and working with masculine codes, values, and motivations. The importance and value of relatable masculine content and design was a prominent feature within the survey and interview data. Herein the design and messaging of the campaign were deemed appealing to men as they resonated with men’s protector and provider identities to legitimize and motivate involvement in suicide prevention. Buddy Up afforded an important vehicle for men’s involvement, offering a sense of purpose, belonging, and community.

#### Leveraging Participation to Initiate Conversations and Promote Mental Health: “It Gives Men Language and License to Start Asking Questions”

Interview participants promoted the campaign within workplaces, health care settings, community centers, and universities, where they engaged with friends, family, colleagues, clients, employees, students, and community members. Kathy, a 65-year-old university administrator, reflected on her experience promoting the campaign to staff and students at a large university:

I think there are intentionally a number of different levels for participation which is highly appealing because of the flexibility. So it’s about encouraging discussions with men about mental health, posing questions with your son, with your classmate, with a co-worker, asking how they’re doing and engaging in conversations. Then there’s material that just gives basic information on the fact that it’s okay to ask and what you might say without feeling like you’re stepping into the role of a therapist.

Similar to Kathy, many interview participants spoke of the need to encourage and initiate conversations about men’s mental health and suicide prevention. Herein, interview participants discussed leveraging their campaign participation in different ways by directly and indirectly discussing mental health with other men. Albert, a 63-year-old retired man, discussed how the campaign afforded him the confidence and clout to inquire and talk about mental health with other men:

I’ve also used it on a personal level just in talking with other men. Informal sort of chit chat, but also, you know, being able to ask the question: “Are you doing okay?” And one of the things I like about the campaign is it gives men language and license. It gives them permission to start asking questions when they see something because oftentimes I think we have gut feelings that something’s not right, and it’s the license then to start asking questions.

Similar to Albert, many interview participants used campaign activities and resources to initiate conversations with men and justify inquiring about their mental health. In this way, the campaign provided an excuse to inquire about mental health and could be used to explain discomforts that might arise during the conversation. Richard, a 70-year-old retired man, discussed how he utilized the Buddy Up Activity Card, a checklist of campaign activities (e.g., “have a coffee with a buddy”), to engage other men in mental health promotion activities and initiate conversations about mental health:

You check off all your activities and it’s encouraging. I sent that on to some other men I know to get involved. When I’ve been doing stuff with them, I show them the sheet, saying, “yeah, we’re out for a walk. I’m checking off something on my sheet because I’m here with you.”

However, while positioning the campaign as a centerpiece in discussions helped some participants to engage in conversations, others were more reticent to directly discuss mental health or disclose their campaign participation. Tom, a 24-year-old university student, expressed his hesitation in disclosing his participation in a suicide prevention campaign to some men:

I think it depends on the group if I’ll mention if it’s with Buddy Up. I think if there is a little bit more resistance, I might be less likely to mention that it’s Buddy Up and approach it as more of just hanging out and exercising together, or whatever the activity is, as opposed to being like, “hey, this is a mental health campaign.”

The tenets of the campaign clearly resonated with Tom as he sought opportunities to actively engage in peer support and mental health promotion. Yet, he carefully considered his approach and was sensitive to the potential for the “mental health” label to deter some men. Instead, Tom provided an example of covert mental health promotion, demonstrating understanding of an approach to doing peer support without naming it. Tom’s approach may also support survey findings that suggest a need for more information on how to initiate difficult conversations with men.

Findings in this theme revealed ways in which participants mechanistically utilized Buddy Up to engage men in suicide prevention. Highlighted were covert strategies that participants used to negotiate and norm peer support for men’s mental health promotion. Participants’ approaches were predicated on understanding men’s receptivity to health-promoting conversations and activities which they used to adapt their delivery.

#### Driving New Masculine Cultures: “We Start Every Meeting With a Mental Health Moment”

Campaign participation was diverse and varied contextually for many participants. Interview participants revealed several creative and unique approaches to men’s mental health promotion and suicide prevention. For example, Mark (35-year-old, unemployed) shared his creative use of the stickers to raise awareness for suicide prevention:

They have stickers with like a little thumb print logo, I actually cut it up and put the thumb print on my right above my outside door handle. My door is orange already, just by coincidence, and I just thought this is going to be a message to guests who come to the house, or anyone who drops off a package, or whatever. This will be just a little heads-up.

For many participants like Mark, there were clear intentions to raise awareness and challenge masculine norms of self-reliance and stoicism. In this way, Buddy Up might be understood as a driver and catalyst for cultural change. Within workplaces, some interview participants suggested Buddy Up was a point-of-entry or pathway to their Employee and Family Assistance Program whereas others discussed procedural and policy changes. Steve, a 34-year-old man involved in his workplace Health and Safety Committee, discussed how Buddy Up encouraged procedural changes that promoted mental health:

We were doing some mental health stuff before, but. . ..it’s becoming more of a topic that people aren’t afraid to discuss as much . . . They want us to start every meeting with a safety moment. So someone will say, “Last night I was putting on my Christmas lights, and I had my extension ladder out, and I asked my wife to come out and hold the ladder because it was pretty wobbly” and everyone says, “oh, great safety moment!” So now we do a mental health moment. If someone said, “on the weekend, I had a buddy having some issues with his wife, and he was concerned that she may be leaving him, and he gave me a shout and asked if I could come out. So I pulled another guy up, and we drove out there and talked with him and reassured him, and got him in the right hands of, say, his dad. That would be like a mental health moment.

Steve discussed the creative adoption and adaptation of Buddy Up with his workplace as a catalyst for change. Similar to Steve, many interview participants believed that they had helped to raise awareness for mental health promotion and suicide prevention and normalize peer discussions and help-seeking. Oliver, a 36-year-old construction manager, discussed how promoting Buddy Up had helped to shift workplace culture and potentially prevent crisis situations:

I had one situation where a guy came up to his foreman and said he’s not doing well. I intercepted him and chatted and we got talking about stuff. The police came in and did a welfare check on him, because we didn’t know at that point what was going to happen. It was a situation where he felt comfortable enough to ask me, “can I text you whenever I’m having any issues?” He’s back to work now . . . I text him every once in a while, and just say, “hey, how’s it going?” I ran into him on a site recently, and he gave me a big bear hug.

However, while many interviewees perceived their promotion of Buddy Up to be meaningful and important, they were universally challenged to identify the reach or impact that their work had on men. When distributing resources or sharing information on social media, participants discussed how difficult it was to measure the impact of their work. David, a 38-year-old man, frequently promoted Buddy Up to organizations through his health service work and discussed difficulties with assessing uptake in these settings:

It’s hard to say how much engagement we’re getting at times, because you can’t be there when people are coming to take resources . . . I forward that email out to my distribution list, encourage everybody to continue these pieces, but nobody actually gets back to me with a group picture that says, “all this was done,” right? That’s kind of the unmeasurable component.

David identified the intangibility and challenges for evaluating cultural change, wherein participants’ efforts were focused on contextually and circumstantially doing “good work,” rather than the empirical evidence of rupturing and reformulating masculine cultures. Herein, the unit of measurement that could be used to meaningfully evaluate cultural impacts might vary greatly by context.

In summary, this theme revealed participants’ buy-in to changing milieus by challenging masculine norms of self-reliance. Here, participants used Buddy Up to support cultural ideals for men’s comradery and peer support through teamwork and crisis prevention actions. Participants were careful to work within masculine and cultural bounds and Buddy Up offered them the legitimacy and cover to do that work.

## Discussion

Findings from this research provide evidence for the feasibility and acceptability of Buddy Up and highlight important factors that may contribute to men’s engagement with suicide prevention campaigns more broadly. Campaign satisfaction was high and participants’ feedback was resoundingly positive. Strengths of the campaign included appealing messaging, straightforward calls-to-action, tangible resources and materials, and flexible delivery options. These findings support mental health promotion strategies identified by others (Robertson et al., 2018; Seidler et al., 2018), and provide important insights into men’s experiences and engagement in suicide prevention campaigns.

Pirkis et al. (2019) identified the need to better understand appealing and effective campaign messaging in their review of suicide prevention media campaigns. Respondents in the present study indicated high levels of acceptability for the framing of Buddy Up, which focused on encouraging men to monitor and provide peer support for men’s mental health challenges. While help-seeking may be perceived as synonymous with weakness or giving up (Seidler et al., 2016), positioning men as the benefactors of support-giving aligns with masculine values and virtues of being a protector and provider. Findings suggest that men were highly receptive to this approach, and many participants indicated benefits through the development of healthy interpersonal relationships and masculine cultures conducive to mental health promotion. While the approach resonated with many men, the realities of engaging in peer support could be challenging. These findings are in line with Chandler (2022) who suggested that it is important to also consider interpersonal and contextual factors that impact men’s ability to communicate emotions and distress. Indeed, some participants requested additional resources and support to comfortably navigate the complexities and bounds of supporting men who were experiencing mental health challenges, particularly in the context of suicide risk. Future research might purposefully examine the gendered aspects of men’s peer support for critical mental health incidents. With regard to campaign development, efforts may be targeted at upskilling and training men how to provide (and receive) peer support for mental health challenges. Specifically, participants spoke of an enduring discomfort or awkwardness around mental health-related conversations that persist despite increased awareness of the importance of mental health promotion. Future campaigns might adopt more covert mental health promotion strategies that reduce the emphasis on language and methodical dialogue (i.e., asking someone how they are doing) as the primary vehicle for connection and peer support.

Participants’ involvement with Buddy Up revealed complex and nuanced approaches to men’s mental health promotion and suicide prevention. Showcased were creative community-based strategies for engaging in peer support which offer insight into how some men foster and maintain healthy supportive relationships that enhance mental health. Participants demonstrated an awareness and understanding of masculine norms and cultures and the influence that restrictive ideals can have on men’s engagement with mental health promotion and suicide prevention. While some focused on interpersonal relationships, others sought to influence cultures more broadly. The complex interplay between masculinities and men’s mental health demands consideration of gender at multiple levels in campaign design and/or tailored intervention content. Many participants in the present study demonstrated a profound awareness and understanding of these complexities by actively working with and around masculine norms to promote men’s mental health. Particularly following the COVID-19 pandemic, the diversity and prevalence of mental health challenges among men suggests the need to carefully consider strategies for addressing gender norms, roles, and relations (Zielke et al., 2023). Findings demonstrated how men’s suicide prevention is inescapably linked to masculinities. The particular resonance of Buddy Up for participants may have been a function of the diverse range of avenues for mental health promotion while leveraging, rather than circumventing, traditional masculinities.

Strengths of the current study include the inductive approach used to synthesize the feasibility and acceptability parameters of Buddy Up and their potential for transferability to guide other similar initiatives. While studies often utilize researcher-derived measures of feasibility (e.g., retention rates, satisfaction ratings), key campaign features identified in the present study were purposefully participant-driven (e.g., relatable male characters, masculine cultural changes). However, due to the self-selected nature of the study sample and proportion of participants with prior suicide prevention and/or health care experience, a limitation is that the findings may not be indicative of all individuals’ experiences. In addition, as participants were recruited from a campaign mailing list, the perspectives of individuals who did not register and provide a contact email may have been omitted. That many participants were women (i.e., Survey: 60%, Interviews: 21%) is also notable and raises important questions about their role in men’s suicide prevention. Women are important allies in men’s health who have long taken on advocacy and caregiving roles. It is important to ensure that traditional femininities are not being perpetuated to compensate (or excuse) unhealthy masculine cultures. Work is still needed to encourage men to adopt active and primary roles in mental health promotion and early intervention. In addition, many participants had prior experience with suicide prevention. Finally, while the diverse contexts in which the campaign was promoted hold promise for broader applications, the challenges for proving attribution of outcomes to specific participation patterns limits the evaluation of Buddy Up.

Men’s suicide prevention campaigns have demonstrated promising results (Struszczyk et al., 2019). As new campaigns emerge, it is important to understand which program aspects resonate with and retain men. Findings from the current study demonstrate the complex ways in which participants engage in and leverage their campaign participation to [re]negotiate masculine identities in promoting men’s mental health.

## Supplemental Material

sj-docx-1-jmh-10.1177_15579883231209189 – Supplemental material for “Hey Amir, How Are You REALLY Doing?”: Participant Perspectives of a Peer-Based Suicide Prevention Campaign for Men Click here for additional data file. Supplemental material, sj-docx-1-jmh-10.1177_15579883231209189 for “Hey Amir, How Are You REALLY Doing?”: Participant Perspectives of a Peer-Based Suicide Prevention Campaign for Men by Paul Sharp, Patricia Zhu, John S. Ogrodniczuk, Zac E. Seidler, Michael J. Wilson, Krista Fisher and John L. Oliffe in American Journal of Men's Health
