# Supplementary material for: “Hey Amir, How Are You REALLY Doing?”: Participant Perspectives of a Peer-Based Suicide Prevention Campaign for Men
Source: Am J Mens Health. 2023 Oct 30;17(5):15579883231209189. doi: 10.1177/15579883231209189 (PMC10619351; doi:10.1177/15579883231209189)
Supplement: sj-docx-1-jmh-10.1177_15579883231209189 – Supplemental material for “Hey Amir, How Are You REALLY Doing?”: Participant Perspectives of a Peer-Based Suicide Prevention Campaign for Men [file sj-docx-1-jmh-10.1177_15579883231209189.docx]

**Appendix A. Interview Guide**

1. How did you hear about Buddy Up?
2. What were the key strengths of the campaign?
3. What are the challenges/weaknesses of the campaign?
4. What was your motivation for participating in Buddy Up?
5. There are a number of different ways to be involved in Buddy Up. Tell me about a notable experience you had.
   1. What was your biggest achievement during the campaign?
6. What did you learn about men’s mental health or suicide prevention?
7. How did you promote the Buddy Up campaign to others?
   1. What did you find most effective?
8. What changes did you observe during the campaign?
9. What unexpected outcomes occurred (positive or negative)?
10. What are your recommendations for improving the campaign?
